# Heterogeneous Activity Profiles in Older Adults: A Latent Class Analysis and Validation With Health Outcomes

**DOI:** 10.7759/cureus.99585

**Published:** 2025-12-18

**Authors:** Keita Nakahara, Yuri Fujii, Nobuyuki Honda, Kohei Kusuda, Tatsuya Yamamoto, Jumpei Oba

**Affiliations:** 1 Department of Rehabilitation, Kansai University of Welfare Sciences, Osaka, JPN; 2 Department of Occupational Therapy, Faculty of Rehabilitation, Kobe Gakuin University, Hyogo, JPN

**Keywords:** activity profile, frailty, latent class analysis (lca), older adults, validation study

## Abstract

Objective

The inconsistent effectiveness of frailty prevention may stem from a research focus on the *quantity* of physical activity while overlooking its qualitative *patterns*. This study aimed to identify distinct activity profiles among community-dwelling older adults in Japan and to validate their association with key health indicators.

Methodology

A cross-sectional web-based survey was conducted with 771 community-dwelling older adults in Japan. Latent Class Analysis (LCA) was applied to data from a 46-item activity checklist, developed by the Japanese Association of Occupational Therapists, to extract distinct activity profiles. The validity of these identified profiles was then examined by analyzing their associations with frailty (using the Kihon Checklist), fear of falling, and various sociodemographic variables.

Results

The analysis identified a four-class model as the optimal solution, revealing four heterogeneous profiles: (1) Inactive and Role-Limited, with the highest frailty risk; (2) Community-Focused and IADL (instrumental activities of daily living)-Imbalanced, predominantly males with low participation in domestic chores; (3) IADL-Proficient and Hobby-Limited, mainly females with limited social engagement; and (4) Lifelong Active and Socially Engaged, with the best health status. These profiles were strongly associated with frailty, fear of falling, age, gender, and socioeconomic status.

Conclusions

The lifestyles of older adults are not monolithic but can be classified into qualitatively distinct patterns that are closely linked to health status. Notably, even among groups with similar total activity levels, differences in activity content reflecting gender roles suggest distinct health risks. Effective frailty prevention requires an approach that focuses on the qualitative *patterns* of real-life circumstances, not just the *quantity* of activity.

## Introduction

In a society with a high proportion of older adults, frailty represents a significant public health concern, as it not only markedly diminishes an individual's quality of life (QoL) but also functions as a primary catalyst for escalating healthcare and long-term care expenditures. In response, the World Health Organization (WHO) defines "healthy aging" as "the process of developing and maintaining the functional ability that enables well-being in older age," thereby positioning frailty as its conceptual opposite [1]. In addressing this issue, support strategies have been investigated primarily through the lens of exercise therapy, and clinical guidelines have strongly recommended interventions focusing on exercise, nutrition, and the review of polypharmacy [2]. However, the effectiveness of these interventions has been inconsistent, and even multicomponent interventions have reported limited effects, with notable variability in outcomes across studies [3]. The phenomenon of "variance in effectiveness" indicates the necessity for a more personalized approach that considers not only "what" the intervention is, but also "to whom" and "how" it is delivered.

We posit that the root cause of these inconsistent outcomes lies in the limitations of two foundational approaches that have traditionally underpinned frailty research. The initial constraint pertains to the "deficit model," which predominantly emphasizes the evaluation of "deficits" in physical and mental function concomitant with the aging process [4,5]. While the model demonstrates clinical utility, it tends to overlook positive aspects that contribute to a rich life in older age, such as social participation and hobbies. This tendency to focus on the physical domain may result in a bias toward interventions that are focused on the physical domain [6].

A more fundamental limitation is the "variable-centered approach" that underpins conventional analytical methods. This approach analyzes the relationship between individual variables, such as "activity duration" or "number of activity types," and health outcomes. However, it fails to capture the qualitative combinations of activities that constitute an individual's lifestyle, that is, their "patterns." The daily lives of older adults are composed of a diverse and dynamic range of activities, encompassing social, domestic, and leisure pursuits [7]. Despite the presence of similar total activity levels, these intricate patterns can exhibit substantial disparities, attributable to individual factors such as gender and residential environment. These disparities may be associated with divergent risks of frailty and cognitive decline [8]. To surmount these structural limitations, this study adopted a "person-centered approach" to identify subgroups of individuals who share similar activity patterns. This approach entails the classification of diverse combinations of activities into "activity profiles," [9] a strategy that fosters a more realistic understanding of the heterogeneity among older adults and suggests pathways for highly individualized support [10].

Therefore, the present study pursued two primary objectives: first, to empirically identify qualitatively distinct activity profiles among community-dwelling older adults in Japan using Latent Class Analysis (LCA), and second, to validate the clinical utility of these identified profiles by examining their associations with key health indicators (including frailty [11], physical activity level [12], and fear of falling [13]) and sociodemographic factors.

## Materials and methods

Participants

The data for this study were collected from registered monitors of a web-based survey panel operated by iBRIDGE Inc. Participants were remunerated for their participation by the online survey company. The target population comprised healthy older adults aged 65 and over residing in Japan. The quota sampling method was implemented in accordance with the age demographics enumerated in the Japanese national census, with a target of 800 responses. The exclusion criterion was individuals certified as needing support or long-term care (*n* = 29) under Japan's national insurance system. This criterion was applied to effectively screen out participants likely experiencing severe functional limitations or marked cognitive impairment, as this certification process assesses both physical and cognitive function. This resulted in 771 valid responses for the final analysis.

Procedure

The present study was conducted as a web-based, cross-sectional questionnaire survey in March of 2025. The research protocol was reviewed and endorsed by the Ethics Review Committee of Kansai University of Welfare Sciences (Approval No. 24-38). Before their participation, all individuals were provided with an online explanation of the study's purpose, the anonymous handling of data, the voluntary nature of their participation, and the fact that no disadvantages would arise from refusal. The protocol was duly approved by the relevant institutional review board, and all participants provided written informed consent via electronic means. The present study was conducted in accordance with the guidelines outlined in the Declaration of Helsinki.

Measures

Activity Profile

The assessment of activity profiles was conducted using the "Interest Checklist," a 46-item tool developed by the Japanese Association of Occupational Therapists (JAOT) as part of the Management Tool for Daily Life Performance (MTDLP) [14]. Permission to use this scale was obtained from the JAOT. Although the original checklist can be used to identify activities a person "wants to do" or is "interested in," the present study adapted the instruction to focus exclusively on actual behavior. Participants were instructed to respond "yes" or "no" to each of the 46 items based solely on whether they were "currently engaged in (doing)" that activity. The subsequent analysis was therefore based on data reflecting participants' actual behavior.

The following external variables were utilized to examine the construct validity of the identified latent classes.

Frailty: The assessment of frailty was conducted using the Kihon Checklist, a tool developed by the Japanese Ministry of Health, Labor, and Welfare (MHLW) for preventive care [15]. This screening tool is designed to comprehensively identify risks of functional decline in older adults. It consists of 25 items across seven domains. Participants were instructed to respond "yes" or "no" to each item. Participants were classified as robust (total score: 0-3), pre-frail (4-7), or frail (≥8) based on previous research.

Physical activity: Physical activity levels were measured using the Japanese short version of the International Physical Activity Questionnaire (IPAQ) [16]. The IPAQ is a self-report questionnaire designed to provide comparable data on health-related physical activity. Participants reported the number of days and minutes spent in vigorous-intensity activity, moderate-intensity activity, and walking during the previous seven days. Based on the IPAQ scoring protocol, physical activity levels were then categorized as low, moderate, or high. The duration of time spent in a seated or reclined position (sedentary time) was also calculated.

Fear of Falling: Fear of falling was assessed using the Japanese version of the Falls Efficacy Scale (FES), originally developed by Tinetti et al. [13]. The version used in this study was an established translation provided by the Japanese MHLW (Appendix). The FES is a tool designed to assess an individual's confidence in performing ten activities of daily living (ADL) without falling. This confidence is measured irrespective of the participant's actual ability to perform the activities. Each item was evaluated using a 4-point scale ranging from 1 ("Not confident") to 4 ("Completely confident"). The total score ranges from 10 to 40 points, with higher scores indicating greater confidence in not falling.

Utilization of Covariates as Predictors of Class Membership

The descriptive statistics were calculated to summarize the baseline characteristics of the study population (*N *= 771). For the continuous variable, age, the mean and standard deviation (SD) were reported. For all categorical variables, including gender, marital status, employment status, income, presence of children, household composition, and education level, frequencies and percentages were reported.

The following is the analysis strategy:

To address the primary objectives stated in the introduction, the analysis was conducted in two primary stages: (1) the identification of latent activity profiles, and (2) the validation of these profiles.

The initial stage of the procedure is the identification of latent profiles and the determination of the number of classes.

The initial step entailed conducting an exploratory LCA with solely the indicator variables to identify profiles based exclusively on activity patterns. A series of models was estimated, with the number of classes increasing sequentially from two to five. The determination of the optimal number of classes was achieved through a triangulation of information from multiple sources, evaluating the Bayesian Information Criterion (BIC), likelihood ratio tests (Lo-Mendell-Rubin likelihood ratio test (LMR LRT) and Vuong-Lo-Mendell-Rubin likelihood ratio test (VLMR LRT)), classification quality (entropy), and class interpretability.

The second stage of the process is the validation of latent profiles, which is achieved through the implementation of the BCH method.

Subsequently, to examine the construct validity of the four identified profiles, we investigated the association between class membership and external variables using the bias-adjusted three-step approach (BCH method) [17]. This approach is designed to address classification errors, thereby facilitating an unbiased estimation of the relationship between the latent profiles and external variables.

The validation process entailed two distinct analyses. First, a multinomial logistic regression analysis was performed to identify sociodemographic predictors of class membership. In this model, the identified latent profiles (four classes) served as the categorical dependent variable, while sociodemographic variables (e.g., age, gender, income) were entered as independent variables (predictors). In order to guarantee statistical stability and facilitate interpretation, the "IADL-Proficient and Hobby-Limited" profile (Class 3) was designated as the reference category. Subsequently, analysis of variance (ANOVA) was employed to validate the profiles' relevance to health outcomes. In this analysis, the four identified profiles were treated as independent variables, and health status and behavioral indicators (e.g., physical activity level, daily sedentary time, frailty, fear of falling) were designated as dependent variables. The analysis involved testing for significant differences in means between profiles. All analyses were conducted using the R (version 4.1.0) and Mplus (version 8.9) software programs.

## Results

Participant characteristics

The final analytical sample consisted of 771 participants. The mean age was 71.3 years (SD = 5.1), with 334 (43.3%) males and 437 (56.7%) females. Detailed demographic characteristics of the participants are presented in Table 1.

**Table 1:** Baseline characteristics of the study population. *n* (%); mean (SD) 1 USD is approximately 147 JPY.

Characteristic		*n* (%) (*N* = 771)
Age (years)		71.3 (5.1)
Gender		
	Male	334 (43%)
	Female	437 (57%)
Marital status		
	Unmarried	169 (22%)
	Married	602 (78%)
Employment status		
	Unemployed	517 (67%)
	Employed	254 (33%)
Income		
	Less than $6,800	53 (6.9%)
	$6,800-$13,600	100 (13%)
	$13,600-$20,400	130 (17%)
	$20,400-$27,200	129 (17%)
	$27,200-$34,000	110 (14%)
	$34,000-$40,800	61 (7.9%)
	$40,800-$47,600	39 (5.1%)
	$47,600-$54,400	38 (4.9%)
	$54,400-$61,200	29 (3.8%)
	$61,200-$68,000	26 (3.4%)
	$68,000-$81,600	20 (2.6%)
	$81,600-$102,000	16 (2.1%)
	$102,000-$122,400	5 (0.6%)
	$122,400-$136,000	9 (1.2%)
	$136,000 or more	6 (0.8%)
Children status		
	Yes	589 (76%)
	No	182(24%)
Household composition		
	Living Alone	145 (19%)
	Living with one other person	441 (57%)
	Living with two or more people	185 (24%)
Education level		
	Completed high school or less	289 (37%)
	Some colleges or higher	482 (63%)

Determining the optimal number of latent classes

Table 2 presents the results of the model comparison for solutions ranging from two to five classes. The Bayesian Information Criterion (BIC) reached its minimum value in the four-class model. Furthermore, the *P*-values for both the LMR and VLMR likelihood ratio tests were statistically significant up to the four-class model but became non-significant for the 5-class model, indicating that increasing to five classes did not improve model fit. The entropy was excellent for both the three-class (0.880) and four-class (0.871) models. Based on a comprehensive assessment, we concluded that the four-class model provided the optimal solution.

**Table 2:** Model selection criteria for competing latent class models. BIC, Bayesian Information Criterion

Evaluation criteria	Model fit (BIC)	Vuong-Lo-Mendell-Rubin	Lo-Mendell-Rubin tests	Entropy
2-class model	28974	<0.001	<0.001	0.816
3-class model	28477	<0.001	<0.001	0.880
4-class model	28242	<0.001	<0.001	0.871
5-class model	28248	0.092	0.093	0.852

Profile characteristics and their predictors

The four latent profiles are detailed below based on their activity participation rates (Table 3) and the sociodemographic predictors identified through multinomial logistic regression (Table 4). Profile 3, the largest class, served as the reference category for the regression analysis.

**Table 3:** Description of the four latent profiles of activity patterns.

No.	Activity	Class 1: Inactive and Role-Limited (*n* = 20), *n* (%)	Class 2: Community-IADL Imbalanced (*n* = 350), *n* (%)	Class 3: IADL-Proficient and Hobby-Limited (*n* = 173), *n* (%)	Class 4: Lifelong Active and Socially Engaged (*n* = 227), *n* (%)
1	Going to the toilet independently	12 (60%)	350 (100%)	173 (100%)	227 (100%)
2	Bathing independently	3 (15%)	342 (98%)	173 (100%)	227 (100%)
3	Dressing oneself	9 (45%)	345 (99%)	172 (99%)	226 (100%)
4	Feeding oneself	3 (15%)	338 (97%)	174 (100%)	225 (99%)
5	Brushing teeth	7 (35%)	344 (98%)	173 (100%)	225 (99%)
6	Grooming	0 (0%)	294 (84%)	172 (99%)	227 (100%)
7	Sleeping as desired	7 (35%)	268 (77%)	159 (91%)	214 (94%)
8	Cleaning	0 (0%)	244 (70%)	174 (100%)	225 (99%)
9	Cooking	4 (20%)	95 (27%)	169 (97%)	188 (83%)
10	Shopping	1 (5%)	303 (87%)	170 (98%)	224 (99%)
11	Home maintenance	2 (10%)	154 (44%)	126 (72%)	200 (88%)
12	Doing laundry	1 (5%)	135 (39%)	174 (100%)	199 (88%)
13	Riding a bicycle/Driving a car	2 (10%)	266 (76%)	111 (64%)	198 (87%)
14	Using public transportation	0 (0%)	160 (46%)	116 (66%)	209 (92%)
15	Caring for grandchildren	1 (5%)	83 (24%)	33 (19%)	113 (50%)
16	Caring for pets	1 (5%)	46 (13%)	21 (12%)	61 (27%)
17	Socializing with friends	2 (10%)	142 (41%)	98 (57%)	214 (94%)
18	Spending time with family/relatives	2 (10%)	246 (70%)	115 (66%)	212 (93%)
19	Dating/Socializing with the opposite sex	0 (0%)	21 (6%)	11 (6%)	42 (19%)
20	Going to an izakaya	0 (0%)	85 (24%)	18 (11%)	113 (50%)
21	Volunteering	0 (0%)	19 (6%)	8 (5%)	72 (32%)
22	Community activities	0 (0%)	65 (19%)	19 (11%)	114 (50%)
23	Visiting shrines/temples	0 (0%)	97 (28%)	46 (26%)	119 (52%)
24	Lifelong learning/studying history	0 (0%)	22 (6%)	9 (5%)	51 (22%)
25	Reading	2 (10%)	121 (35%)	79 (46%)	191 (84%)
26	Haiku	0 (0%)	0 (0%)	0 (0%)	8 (4%)
27	Calligraphy (Shodo)	0 (0%)	0 (0%)	2 (1%)	22 (10%)
28	Drawing/picture letters	0 (0%)	4 (1%)	3 (2%)	24 (11%)
29	Using a computer/word processing	4 (20%)	314 (90%)	125 (72%)	205 (90%)
30	Photography	0 (0%)	56 (16%)	30 (17%)	123 (54%)
31	Movies/theater/concerts	0 (0%)	106 (30%)	49 (28%)	167 (74%)
32	Tea ceremony/Ikebana	0 (0%)	6 (2%)	6 (3%)	33 (15%)
33	Singing/Karaoke	1 (5%)	62 (18%)	17 (10%)	96 (42%)
34	Music/instrument	0 (0%)	119 (34%)	76 (43%)	171 (75%)
35	Board/table games	0 (0%)	65 (19%)	8 (5%)	53 (24%)
36	Gymnastics/Exercise	0 (0%)	75 (22%)	56 (32%)	150 (66%)
37	Walking/strolling	3 (15%)	186 (53%)	94 (54%)	202 (89%)
38	Sports	0 (0%)	45 (13%)	3 (2%)	53 (24%)
39	Dancing	2 (10%)	4 (1%)	4 (2%)	21 (9%)
40	Watching sports	1 (5%)	121 (35%)	29 (17%)	101 (45%)
41	Gambling	1 (5%)	44 (13%)	7 (4%)	21 (9%)
42	Knitting/Crocheting	2 (10%)	0 (0%)	13 (8%)	29 (13%)
43	Needlework	1 (5%)	5 (2%)	45 (26%)	81 (36%)
44	Farming	1 (5%)	44 (13%)	12 (7%)	50 (22%)
45	Employment	1 (5%)	79 (23%)	31 (18%)	89 (39%)
46	Traveling/hot springs	7 (35%)	180 (52%)	89 (51%)	198 (87%)

Profile 1

**Table 4:** Results of multinomial logistic regression predicting latent class membership. *P* < 0.05, **P < 0.01, ***P < 0.001. Statistically significant results are shown in bold. The reference category is Class 3.

Class	Inactive and Role-Limited Type vs. IADL-Proficient and Hobby-Limited Type			Community-IADL Imbalanced Type and IADL-Proficient and Hobby-Limited Type			Lifelong Active and Socially Engaged Type vs. IADL-Proficient and Hobby-Limited Type		
Predictor	Odds ratio (OR)	95% confidence interval (CI)	*P*-value	Odds ratio (OR)	95% CI	*P*-value	Odds ratio (OR)	95% CI	*P*-value
Age	1.011	(0.965, 1.060)	0.648	0.989	(0.947, 1.033)	0.622	1.063	(1.016, 1.113)	0.009**
Gender (Male)	19.015	(11.156, 32.412)	<0.001***	0.92	(0.568, 1.488)	0.733	3.425	(2.137, 5.489)	<0.001***
Marital Status (Widowed/Divorced)	0.448	(0.217, 0.923)	0.029*	0.773	(0.454, 1.315)	0.343	0.582	(0.302, 1.123)	0.106
Job Status (Unemployed)	0.705	(0.410, 1.211)	0.205	0.961	(0.599, 1.541)	0.869	0.416	(0.252, 0.687)	<0.001***
Household Income	1.032	(0.940, 1.134)	0.505	1.09	(1.004, 1.185)	0.041*	1.106	(1.016, 1.205)	0.021*
Has Children	0.643	(0.345, 1.199)	0.165	0.83	(0.510, 1.351)	0.455	0.799	(0.443, 1.442)	0.457
Household Size	1.721	(1.101, 2.688)	0.017*	0.69	(0.466, 1.020)	0.063	1.548	(1.013, 2.364)	0.043*
Education Level	1.214	(0.780, 1.889)	0.39	1.344	(0.926, 1.952)	0.12	1.947	(1.243, 3.047)	0.004**

Inactive and Role-Limited (*n* = 20, 3%): This class is characterized by remarkably low activity implementation rates across all domains. Except for basic ADL like "Going to the toilet independently" (*n *= 12, 60%), engagement in most IADL, social participation, and hobbies was nearly non-existent. Compared to the reference "IADL-Proficient and Hobby-Limited" profile, being male (odds ratio (OR) = 19.02) and having a larger household size (OR = 1.72) significantly increased the odds of belonging to this class. Conversely, being widowed or divorced significantly decreased the odds (OR = 0.45).

Profile 2

Community-IADL Imbalanced (*n* = 350, 45%): This class demonstrates high implementation rates for activities essential for independent living outside the home, such as "Shopping" (*n* = 303, 87%) and "Driving a car" (*n* = 266, 76%). However, their participation in domestic tasks like "Cooking" (*n* = 95, 27%) is notably lower than in other active profiles. The analysis of predictors showed that a higher household income was the only factor slightly, yet significantly, associated with a higher likelihood of being in this class (OR = 1.09) compared to the reference profile.

Profile 3

IADL-Proficient and Hobby-Limited (*n* = 173, 22%): This class, serving as the reference group, is distinguished by extremely high proficiency in household roles, with nearly all members performing "Cleaning" (*n* = 173, 100%) and "Cooking" (*n* = 169, 97%). However, their engagement in social participation ("Socializing with friends," *n* = 98, 57%) and diverse hobbies ("Volunteering," *n* = 8, 5%) was limited, suggesting a "narrow interest" profile focused on the home.

Profile 4

Lifelong Active and Socially Engaged (*n* = 227, 29%): This class exhibits the highest activity rates across almost all domains, reflecting a highly active and multifaceted lifestyle. Members are extensively involved in social, community, and self-fulfillment activities. Compared to the reference profile, members of this class were significantly more likely to be older (OR = 1.06), male (OR = 3.42), employed (OR for unemployed = 0.42), have a higher household income (OR = 1.11), a larger household size (OR = 1.55), and a higher level of education (OR = 1.95).

Validation of latent profiles with external variables

The four classes exhibited distinct and theoretically consistent profiles on external health indicators (Table 5).

**Table 5:** Validation of latent classes using external health and behavioral variables. IPAQ score: 1, Low; 2, Moderate; 3, High. Higher scores indicate greater physical activity. Kihon Checklist score: Higher scores indicate a higher risk of frailty. FES score: Higher scores indicate greater confidence in performing activities without falling (i.e., lower fear of falling). CI, confidence interval; FES, Falls Efficacy Scale

Indicator variable	Inactive and Role-Limited Type (*n* = 20), mean (95% CI)	The Community-IADL Specialized Type (*n* = 350), mean (95% CI)	The Domestically Proficient, Narrow-Interest Type (*n* = 173), mean (95% CI)	Lifelong Active and Socially Engaged Type (*n* = 227), mean (95% CI)
Physical activity level (IPAQ, 1-3 scale)	1.34 (1.19-1.49)	1.77 (1.65-1.90)	1.68 (1.60-1.76)	2.08 (1.99-2.17)
Sitting time (hours/day)	3.47 (2.21-4.74)	5.94 (5.22-6.67)	5.11 (4.69-5.52)	4.68 (4.25-5.10)
Frailty score (Kihon Checklist, 0-25 scale)	7.17 (5.85-8.50)	4.15 (3.66-4.65)	4.24 (3.92-4.57)	2.89 (2.52-3.26)
Fear of falling (FES score, 10-40 scale)	13.36 (11.64-15.09)	11.34 (10.87-11.81)	11.37 (11.09-11.65)	11.06 (10.72-11.39)

Physical Activity

A clear gradient was observed, with the "Lifelong Active" class having the highest activity level and the "Inactive" class the lowest.

Sitting Time

The "Community-IADL Imbalanced" class reported the longest daily sitting time (*M* = 5.94 hours), while the "Inactive" class reported the shortest (*M* = 3.47 hours).

Frailty

Frailty scores aligned perfectly with the activity classifications. The "Inactive" class had the highest level of frailty (*M* = 7.17), while the "Lifelong Active" class had the lowest (*M* = 2.89).

Fear of Falling

Fear of falling was most pronounced in the "Inactive" class (*M* = 13.36), which was significantly higher than the other three groups.

## Discussion

This study adopted a person-centered research strategy to investigate the activity profiles of older adults in Japan. This strategy was employed to overcome the limitations of the "deficit model" and "variable-centered approach" that are prevalent in conventional frailty research. In line with our first objective, LCA was utilized as the statistical method to identify heterogeneous activity profiles, resulting in four statistically and theoretically robust classes. In line with our second objective, the clinical utility of these profiles was validated. This validation was achieved by examining their associations with both key health indicators (including frailty and fear of falling) and a distinct set of demographic and social background factors. In this discussion, we interpret these findings in line with our research questions and argue for the academic and practical significance of our approach by comparing our results with prior research.

Interpretation of Class 4 (Lifelong Active and Socially Engaged) and Comparison with Prior Research. Class 4 aligns with the conceptual frameworks of successful aging [18] and active aging [19]. These frameworks emphasize multidimensional well-being, including psychological and social aspects. These concepts posit that social participation and engagement in diverse hobbies are critical components [20]. The "Lifelong Active and Socially Engaged" cluster, which was characterized by high social engagement and a wide range of activities, corroborates this theory. Moreover, extant research has demonstrated that groups with high frequencies of social participation tend to be more highly educated and employed [21], which is consistent with the demographic characteristics of this cluster. A particularly noteworthy finding is that, in addition to social participation, this cluster demonstrated the greatest diversity in hobby engagement. This finding is of particular significance given the established efficacy of hobbies in maintaining or enhancing cognitive function [22] and preventing frailty [23] in community-dwelling older adults, as evidenced by numerous studies. The present study identified another salient feature of this cluster: continued employment has been linked to better cognitive function [24] and a lower risk of frailty. Consequently, the "Lifelong Active and Socially Engaged" older adults identified in our study can be regarded as exemplifying a tangible form of positive aging. Their multifaceted engagement across social, recreational, and occupational domains provides integrated support for existing evidence that such lifestyles are strongly associated with the maintenance of cognitive function and the prevention of frailty.

The IADL-Proficient and Hobby-Limited Type (Class 3) represents a "home-centric" lifestyle, characterized by near-perfect implementation rates for domestic chores such as cleaning, laundry, and cooking (Table 3). In contrast, participation in hobbies and social activities is limited. This activity pattern is consistent with numerous reports indicating the persistence of gendered division of labor into old age. It is further inferred that this class is predominantly composed of women relative to the other classes. However, this aptitude in domestic tasks may obscure an underlying future health risk. Guarnera et al. have elucidated from numerous studies that both "social isolation," referring to the objective separation from others, and "loneliness," a subjective feeling, are associated with cognitive decline and an increased risk of dementia [25]. In consideration of the aforementioned framework, the lifestyle of Class 3, characterized by its restricted social interaction, is directly associated with the phenomenon of "social isolation." Moreover, a salient finding from research in this area indicates that the pivotal factor in preserving IADL functionality in women is not the execution of domestic tasks per se, but rather the engagement in a multifaceted array of social activities [26]. This phenomenon may be elucidated by the findings of Okamoto et al., whose study on older Japanese adults indicated that cognitive decline in women is intricately linked to factors such as a shorter educational history and having "domestic work" as their primary occupation [27]. The findings, when considered collectively, suggest that in the event of social isolation among older adults in Class 3, there may be a contraction in their lives to the domestic sphere. This contraction may be associated with an increased vulnerability to psychological and cognitive health problems, such as depression and cognitive decline, via biological mechanisms like a chronic stress response (e.g., cortisol secretion).

Conversely, the Community-IADL Imbalanced Type (Class 2) demonstrates high implementation rates for out-of-home IADLs, such as driving and shopping, but exhibits notably low engagement in domestic chores (Table 3). A particularly noteworthy finding is that no statistically significant gender difference was observed between this class and Class 3 (Table 4). This finding is of significant importance, as it suggests the existence of a group that, irrespective of gender, exhibits proficiency in activities outside the home yet demonstrates a deficit in domestic roles. The most significant risk confronting this class is its prolonged sedentary time, as evidenced by the findings presented in Table 5. While activities such as driving and using a personal computer may appear to be functional, they are often part of a sedentary lifestyle, which is widely considered a significant health risk. Indeed, research findings indicate that prolonged periods of sedentary behavior are associated with an elevated risk of mortality, independent of physical activity levels [28]. This study also demonstrated that this risk is further exacerbated in individuals with cardiometabolic diseases, implying that older adults in Class 2 with such comorbidities may face a severely compounded threat to their life expectancy. Moreover, the absence of engagement in domestic tasks, such as housework, is associated with the forfeiture of opportunities for non-exercise activity thermogenesis (NEAT). It has been established that "heavy housework" is associated with the prevention of frailty in men, while "light housework" is linked to a reduced mortality rate in women. Consequently, non-participation in domestic chores may result in the forfeiture of these gender-specific physical benefits. Although Class 2 currently exhibits a similar level of frailty to Class 3 (Table 5), it is hypothesized that their dual risks of prolonged sedentary time and lack of NEAT may place them at a higher risk for future physical frailty and lifestyle-related diseases.

The Inactive and Role-Limited Type (Class 1) constitutes the most health-compromised group, exhibiting the highest frailty score (Table 5) and characterized by a lack of IADL and social participation. This finding aligns with the existing body of literature that demonstrates a strong correlation between declining physical activity and a marked increase in frailty risk [29]. The observed phenomenon suggests that these individuals may be entrapped within a pernicious cycle of frailty. Consequently, this high-risk group should be accorded a high level of priority for preventive interventions. The predictors for this class are particularly revealing. Contrary to popular belief, factors significantly associated with this high-risk profile were being male (OR 19.015) and having a larger household size (Table 4). This elevated risk among men may be a manifestation of the profound psychosocial impact of losing one's primary occupational role upon retirement. Indeed, a review by Meng et al. suggests that retiring from jobs with complex interpersonal demands can precipitate negative psychological consequences through a loss of attachment to one's occupation [30]. Additionally, residing with family members may engender a "care paradox," wherein the activities of daily living are performed for the individual, thereby reducing their opportunities to remain active. The study's findings revealed an interesting observation regarding sitting time (Table 5): the "Inactive and Role-Limited" class (Class 1) exhibited the shortest duration of sitting time among all classes. This phenomenon does not necessarily indicate elevated levels of physical activity; rather, it may be a distressing indicator of severe physical weakness, suggesting that individuals may have limited physical capacity to even maintain a seated position. This condition could be interpreted as a state of profound functional decline, wherein a significant portion of the day is spent in a recumbent position, and even sedentary intellectual activities are challenging to perform.

It is imperative to acknowledge the limitations of the present study. First, the cross-sectional design precludes causal conclusions between activity profiles and health outcomes; longitudinal studies are necessary to track profile evolution. Second, a significant limitation arises from the use of a web-based survey, which introduces an inherent selection bias known as the "Digital Divide." It has been demonstrated that participants in online panels tend to exhibit higher levels of digital literacy, cognitive function, and socioeconomic status in comparison to the broader older population. This bias is likely to be the reason for the unexpectedly low prevalence of the "Inactive and Role-Limited" group (Class 1, 3%). In the general population, the proportion of such inactive or frail individuals is estimated to be significantly higher. Consequently, the prevalence of specific classes should be interpreted with caution, as they likely underestimate the population of vulnerable older adults who lack internet access. Third, the absence of a mechanism to detect careless responses may have ramifications for reliability. Fourth, given that all measures were self-reported, there is a possibility of recall and social desirability biases. The activity checklist was developed in Japan, and its cross-cultural applicability requires further validation.

## Conclusions

The lifestyles of older adults can be classified into diverse patterns, each of which is linked to physical health outcomes. This research strongly suggests that focusing not merely on the "quantity" of activity but on its "qualitative patterns" may offer a valuable perspective in designing future preventive strategies to combat frailty and promote healthy longevity.

## Figures and Tables

**Figure 1:**
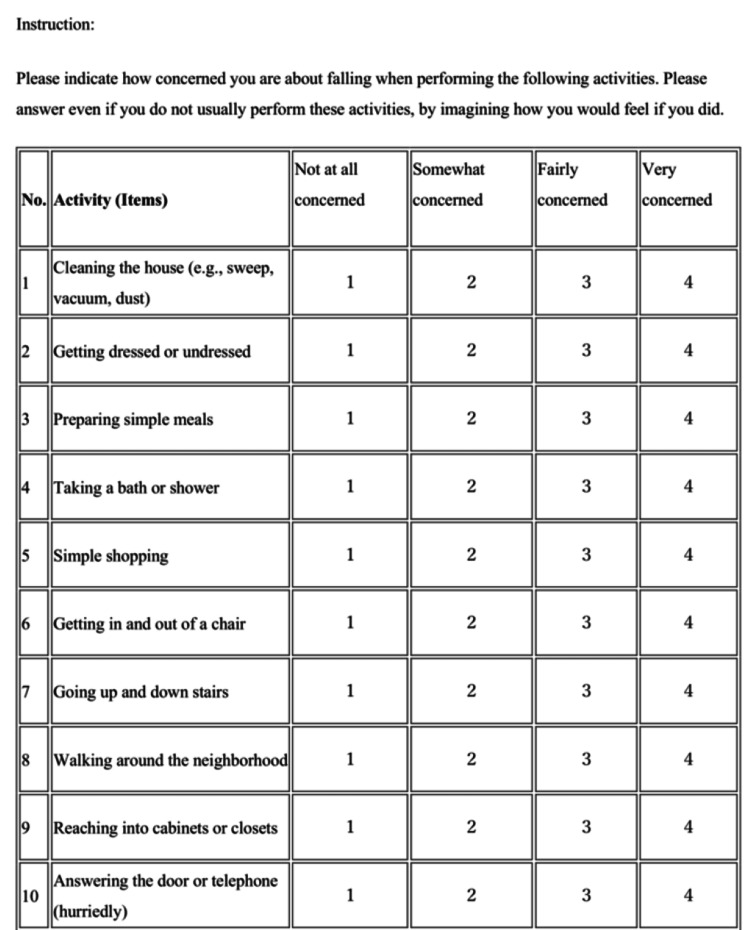
English Translation of the Japanese Modified Falls Efficacy Scale This scale is a modified version of the Falls Efficacy Scale (FES) adopted by the Japanese Ministry of Health, Labour and Welfare. It consists of the original 10 items developed by Tinetti et al. (1990) but utilizes a 4-point Likert scale (1 = Not at all concerned, 4 = Very concerned) instead of the original 10-point scale. The total score ranges from 10 to 40, with higher scores indicating a greater fear of falling (lower falls efficacy).

## Appendices

Appendix 

## References

[1] (2025). World Health Organization: Decade of Healthy Ageing: Baseline Report. Geneva, Switzerland: World Health Organization. https://www.who.int/publications/i/item/9789240017900.

[2] Dent E, Lien C, Lim WS (2017). The Asia-Pacific clinical practice guidelines for the management of frailty. J Am Med Dir Assoc.

[3] Apóstolo J, Cooke R, Bobrowicz-Campos E (2018). Effectiveness of interventions to prevent pre-frailty and frailty progression in older adults: a systematic review. JBI Database System Rev Implement Rep.

[4] Fried LP, Tangen CM, Walston J (2001). Frailty in older adults: evidence for a phenotype. J Gerontol A Biol Sci Med Sci.

[5] Rockwood K, Song X, MacKnight C, Bergman H, Hogan DB, McDowell I, Mitnitski A (2005). A global clinical measure of fitness and frailty in elderly people. CMAJ.

[6] Caicedo-Pareja M, Espinosa D, Jaramillo-Losada J, Ordoñez-Mora LT (2024). Physical exercise intervention characteristics and outcomes in frail and pre-frail older adults. Geriatrics (Basel).

[7] Litwin H, Stoeckel KJ (2016). Social network, activity participation, and cognition: a complex relationship. Res Aging.

[8] Tomioka K, Kurumatani N, Hosoi H (2017). Association between social participation and 3-year change in instrumental activities of daily living in community-dwelling elderly adults. J Am Geriatr Soc.

[9] Todd M, Adams MA, Kurka J (2016). GIS-measured walkability, transit, and recreation environments in relation to older Adults' physical activity: a latent profile analysis. Prev Med.

[10] Grewal I, Lewis J, Flynn T, Brown J, Bond J, Coast J (2006). Developing attributes for a generic quality of life measure for older people: preferences or capabilities?. Soc Sci Med.

[11] Ito K, Kawai H, Tsuruta H, Obuchi S (2021). Predicting incidence of long-term care insurance certification in Japan with the Kihon Checklist for frailty screening tool: analysis of local government survey data. BMC Geriatr.

[12] Craig CL, Marshall AL, Sjöström M (2003). International physical activity questionnaire: 12-country reliability and validity. Med Sci Sports Exerc.

[13] Tinetti ME, Richman D, Powell L (1990). Falls efficacy as a measure of fear of falling. J Gerontol.

[14] (2025). Japanese Occupational Therapist Association. Seikatsukoui koujou manejimento - kyoumi kanshin chekkushiito [Life activity improvement management - interest/concern checklist]. https://www.jaot.or.jp/files/news/wp-content/uploads/2014/05/seikatsukoui-2kyoumikanshin-checksheet.pdf.

[15] Satake S, Senda K, Hong YJ (2016). Validity of the Kihon Checklist for assessing frailty status. Geriatr Gerontol Int.

[16] Hagströmer M, Oja P, Sjöström M (2006). The International Physical Activity Questionnaire (IPAQ): a study of concurrent and construct validity. Public Health Nutr.

[17] Asparouhov T, Muthén B (2014). Auxiliary variables in mixture modeling: three-step approaches using Mplus. Struct Equ Modeling.

[18] Rowe JW, Kahn RL (1997). Successful aging. Gerontologist.

[19] (2025). World Health Organization. Active Ageing: A Policy Framework. https://iris.who.int/bitstream/handle/10665/67215/WHO_NMH_NPH_02.8.pdf.

[20] Gaviano L, Pili R, Petretto AD, Berti R, Carrogu GP, Pinna M, Petretto DR (2024). Definitions of ageing according to the perspective of the psychology of ageing: a scoping review. Geriatrics (Basel).

[21] van Hees SG, van den Borne BH, Menting J, Sattoe JN (2020). Patterns of social participation among older adults with disabilities and the relationship with well-being: A latent class analysis. Arch Gerontol Geriatr.

[22] Lv G, Zhang Y, Liu S (2025). Effect of leisure activities on cognitive and memory function in older adults: A systematic review and meta-analysis of randomized controlled trials. J Clin Neurosci.

[23] Viola E, Martorana M, Ceriotti D, De Vito M, De Ambrosi D, Faggiano F (2024). The effects of cultural engagement on health and well-being: a systematic review. Front Public Health.

[24] Akaida S, Katayama O, Yamaguchi R, Yamagiwa D, Shimada H (2025). Occupational gaps and mild cognitive impairment among older workers. Eur Geriatr Med.

[25] Guarnera J, Yuen E, Macpherson H (2023). The impact of loneliness and social isolation on cognitive aging: a narrative review. J Alzheimers Dis Rep.

[26] Tomioka K, Kurumatani N, Hosoi H (2017). Age and gender differences in the association between social participation and instrumental activities of daily living among community-dwelling elderly. BMC Geriatr.

[27] Okamoto S, Kobayashi E, Murayama H, Liang J, Fukaya T, Shinkai S (2021). Decomposition of gender differences in cognitive functioning: National Survey of the Japanese elderly. BMC Geriatr.

[28] Boucham M, Salhi A, El Hajji N, Gbenonsi GY, Belyamani L, Khalis M (2024). Factors associated with frailty in older people: an umbrella review. BMC Geriatr.

[29] Meng A, Nexø MA, Borg V (2017). The impact of retirement on age related cognitive decline - a systematic review. BMC Geriatr.

[30] Koyama T, Ozaki E, Kuriyama N (2021). Effect of underlying cardiometabolic diseases on the association between sedentary time and all-cause mortality in a large Japanese population: a cohort analysis based on the J-MICC study. J Am Heart Assoc.

